# Antithrombotic prophylaxis following total hip arthroplasty: a level I Bayesian network meta-analysis

**DOI:** 10.1186/s10195-023-00742-2

**Published:** 2024-01-09

**Authors:** Filippo Migliorini, Nicola Maffulli, Erlis Velaj, Andreas Bell, Daniel Kämmer, Frank Hildebrand, Ulf Krister Hofmann, Jörg Eschweiler

**Affiliations:** 1 Department of Orthopaedic, Trauma, and Reconstructive Surgery, RWTH University Medical Centre, Pauwelsstraße 30, 52074 Aachen, Germany; 2 Department of Orthopedics and Trauma Surgery, Academic Hospital of Bolzano (SABES-ASDAA), Teaching Hospital of the Paracelsus Medical University, 39100 Bolzano, Italy; 3 Department of Orthopaedic and Trauma Surgery, Eifelklinik St.Brigida, 52152 Simmerath, Germany; 4 Department of Medicine and Psychology, University of Rome “La Sapienza”, Rome, Italy; 5 School of Pharmacy and Bioengineering, Keele University Faculty of Medicine, Stoke on Trent, ST4 7QB UK; 6 Centre for Sports and Exercise Medicine, Barts and the London School of Medicine and Dentistry, Mile End Hospital, Queen Mary University of London, London, E1 4DG UK; 7 Department of Orthopaedic, Trauma, and Reconstructive Surgery, BG Klinikum Bergmannstrost Halle, Halle (Saale), Germany

**Keywords:** Total hip arthroplasty, Antithrombotic prophylaxis, Deep vein thrombosis, Pulmonary embolism, Haemorrhages

## Abstract

**Background:**

Several clinical investigations have compared different pharmacologic agents for the prophylaxis of venous thromboembolism (VTE). However, no consensus has been reached. The present investigation compared enoxaparin, fondaparinux, aspirin and non-vitamin K antagonist oral anticoagulants (NOACs) commonly used as prophylaxis following total hip arthroplasty (THA). A Bayesian network meta-analysis was performed, setting as outcomes of interest the rate of deep venous thrombosis (DVT), pulmonary embolism (PE) and major and minor haemorrhages.

**Methods:**

This study was conducted according to the Preferred Reporting Items for Systematic Reviews and Meta-Analyses (PRISMA) extension statement for reporting systematic reviews incorporating network meta-analyses of healthcare interventions. All randomised controlled trials (RCTs) comparing two or more drugs used for the prophylaxis of VTE following THA were accessed. PubMed, Web of Science and Google Scholar databases were accessed in March 2023 with no time constraint.

**Results:**

Data from 31,705 patients were extracted. Of these, 62% (19,824) were women, with age, sex ratio, and body mass index (BMI) being comparable at baseline. Apixaban 5 mg, fondaparinux, and rivaroxaban 60 mg were the most effective in reducing the rate of DVT. Dabigatran 220 mg, apixaban 5 mg, and aspirin 100 mg were the most effective in reducing the rate of PE. Apixaban 5 mg, ximelagatran 2 mg and aspirin 100 mg were associated with the lowest rate of major haemorrhages, while rivaroxaban 2.5 mg, apixaban 5 mg and enoxaparin 40 mg were associated with the lowest rate of minor haemorrhages.

**Conclusion:**

Administration of apixaban 5 mg demonstrated the best balance between VTE prevention and haemorrhage control following THA.

*Level of evidence* Level I, network meta-analysis of RCTs.

## Introduction

Hip osteoarthritis is a common cause of pain and disability [1–4]. In patients with advanced osteoarthritis, total hip arthroplasty (THA) is commonly recommended [3, 5–7]. THA demonstrated very good outcomes, improving patient quality of life and participation in recreational activities [8–14]. Although prophylaxis is recommended in all patients, symptomatic venous thromboembolism (VTE) occurs in approximately 2% of patients following lower limb arthroplasty [15–18]. In most patients, VTE may present clinically as deep vein thrombosis (DVT) or pulmonary embolism (PE) [19, 20], both of which are associated with an increased risk of disability and mortality [21, 22]. Previous VTE, varicosities, congestive heart failure, older age, female sex, higher BMI, bilateral surgery, history of prior VTE, surgical time greater than 3.5 h, factor V Leiden, antithrombin and prothrombin gene mutation, oestrogen replacement therapy, traumas and autoimmune disease such as anti-phospholipid syndrome are risk factors for VTE [23–31].

A 4–6 weeks, prophylaxis is recommended to reduce the risk of VTE following primary THA [32–35]. Furthermore, VTE prophylaxis increases the risk of postoperative haemorrhage [36]. Several clinical investigations have compared different types of prophylaxis and protocols for the prevention of VTE [16, 37–49]; however, no consensus has been reached. In a previous Bayesian network meta-analysis including 35 RCTs (53,787 patients), all anticoagulant drugs showed some effectiveness for VTE prophylaxis in total knee and hip arthroplasty, with fondaparinux and rivaroxaban being the most effective [50]. Cohen et al. [51] conducted a meta-analysis on 43 RCTs to assess the efficacy and safety of apixaban versus other anticoagulants in total knee and hip arthroplasty; apixaban, rivaroxaban, and dabigatran demonstrated similar or improved efficacy and similar safety [51].

A Bayesian network meta-analysis was conducted to compare the rate of DVT, PE and major and minor haemorrhages to identify the optimal compound as prophylaxis following THA. The compounds of interest were enoxaparin, fondaparinux, aspirin and non-vitamin K antagonist oral anticoagulants (NOACs).

## Methods

### Eligibility criteria

All randomised controlled trials (RCT) comparing two or more pharmacological modalities of prophylaxis of VTE following THA were accessed. Given the author language capabilities, studies in English, German, Italian, French, and Spanish were considered. Only RCTs with level I evidence, according to the Oxford Centre of Evidence-Based Medicine [52], were considered. Opinions, reviews, editorials, posters, abstracts, comments, and letters were excluded, as were animal, in vitro, and computational investigations. Studies evaluating arthroplasty in other locations were not considered. Studies which reported data on primary THA and/or revision setting in elective and/or emergency (e.g. following femoral neck fracture) surgery were considered. For studies which evaluated total joint arthroplasty in more areas, only the data from THA were collected. Studies evaluating patients who had undergone experimental surgeries or physiotherapeutic protocols were not considered. Missing quantitative data under the outcomes of interests warranted the exclusion of the study.

### Search strategy

This study was conducted according to the 2015 PRISMA Extension Statement for Reporting of Systematic Reviews Incorporating Network Meta-Analyses of Health Care Interventions [53]. The Problem, Intervention, Comparison, Outcomes, Design (PICOD) algorithm was followed:

- P (Problem): VTE in THA;
- I (Intervention): Pharmacological prophylaxis;
- C (Comparison): Apixaban, aspirin, dabigatran, edoxaban, enoxaparin, fondaparinux, rivaroxaban, ximelagatran;
- O (Outcomes): DVT, PE, major and minor haemorrhages.
- D (Design): Randomised controlled trial.

PubMed, Web of Science, and Google Scholar databases were accessed in May 2023 with no time constraint. The following keywords were used in each database for the search using the Boolean operator AND/OR: *hip* AND *arthroplasty* OR *replacement* AND *prophylaxis* OR *prevention* AND *thrombosis* OR *thromboembolism* OR *pulmonary embolism* OR *deep vein thrombosis* OR *embolism* OR *bleeding* OR *haemorrhages* AND *rivaroxaban* OR *aspirin* OR *enoxaparin* OR *anticoagulant* OR *dabigatran* OR *edoxaban* OR *apixaban* OR *direct thrombin inhibitor* OR *fondaparinux* OR *NOACs* OR *non-vitamin K antagonist oral anticoagulants*. The search was restricted to RCTs.

### Data collection and extraction

Two authors (J.E. and E.V.) performed the data selection and collection. The resulting titles from the database searches were screened by hand. If the title matched the topic, the abstract was accessed. If the abstract matched the topic, the full manuscript was accessed. The bibliography of the full-text articles was also screened by hand. All resulting full texts of the articles of interest were downloaded. Both authors compared the articles resulting from the search and controversies were settled by a third author (F.M.). Data extraction was performed by a single author (E.V). in Microsoft Office Excel version 16.71 (Microsoft Corporation, Redmond, USA). Data concerning the following generalities of the included studies were extracted: name of the first author, year and journal of publication, and length of the follow-up (months). Moreover, the following patient demographics were retrieved: mean age, sex ratio, and mean BMI (kg/m^2^). Data concerning the following drugs were extracted: apixaban, aspirin, dabigatran, edoxaban, enoxaparin, fondaparinux, rivaroxaban and ximelagatran. With respect to the outcomes of interest, the following parameters were collected at the last follow-up: DVT, PE and major and minor haemorrhages.

### Assessment of the risk of bias and quality of the recommendations

The risk of bias was evaluated in accordance with the guidelines highlighted in the Cochrane Handbook for Systematic Reviews of Interventions [54]. The risk of bias of the software Review Manager 5.3 (The Nordic Cochrane Collaboration, Copenhagen) was used. The risk of bias evaluation was conducted by two authors (J.E. and E.V.) separately. The following risks of biases were evaluated: selection, detection, performance, reporting, attrition and other biases.

### Synthesis methods

The statistical analyses were performed by the main author (F.M.) following the recommendations of the Cochrane Handbook for Systematic Reviews of Interventions [55]. Baseline demographics were assessed through the IBM SPSS software. Mean and standard deviation were used for continuous variables, and frequency (events/observations) for binary endpoints. Analysis of variance (ANOVA) was used to assess baseline comparability, with values of *P* > 0.1 considered as satisfactory. The network meta-analyses were conducted using the STATA Software/MP, version 14.1 (StataCorporation, College Station, Texas, USA). The STATA routine for Bayesian hierarchical random-effects model analysis was used. The log odds ratio (LOR) effect measure was adopted for analysis of dichotomic data. The overall inconsistency was evaluated through the equation for global linearity via the Wald test. In *P*-values of > 0.5, the null hypothesis could not be rejected, and the consistency assumption could be accepted at the overall level of each treatment. Both confidence (CI) and percentile (PrI) intervals were set at 95%. Edge and interval plots were obtained and evaluated. The funnel plot of each comparison was performed to assess data dispersion.

## Results

### Search result

**Fig. 1:**
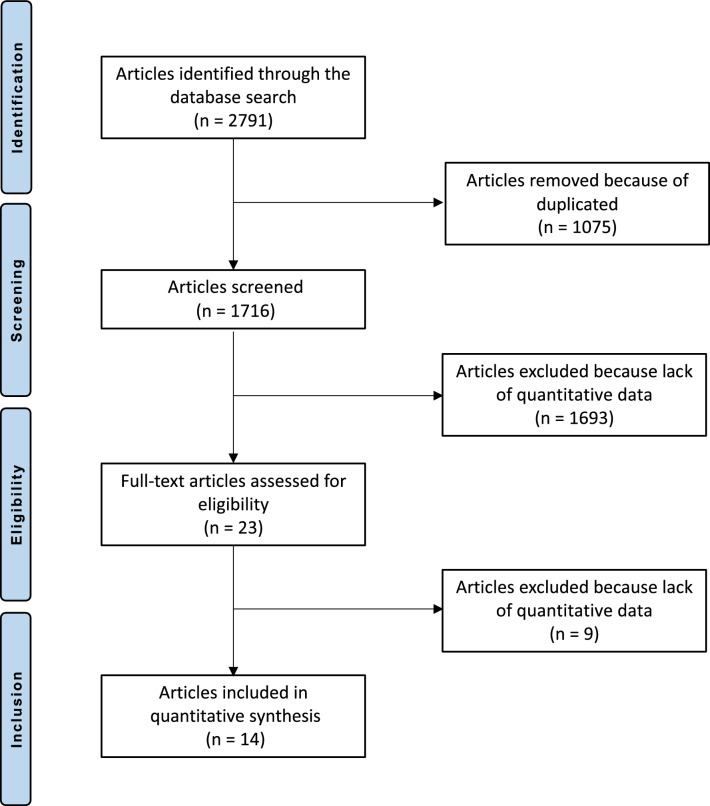
PRISMA flow diagram of the search result

The literature search resulted in 2791 studies. Of them, 1075 were excluded as they were duplicates. An additional 1693 studies were excluded as they did not match the eligibility criteria: not matching the topic (*N* = 1054), type of study (*N* = 423), not focussed on THA (*N* = 175), not reporting data for each outcome separately (*N* = 31), experimental surgeries or physiotherapeutic protocols (*N* = 9) and language limitation (*N* = 1). Additionally, nine studies were excluded as they did not report quantitative data under the outcomes of interest (*N* = 11). Finally, 14 RCTs were included in the present Bayesian network meta-analysis (Fig. 1).

### Risk of bias assessment

**Fig. 2:**
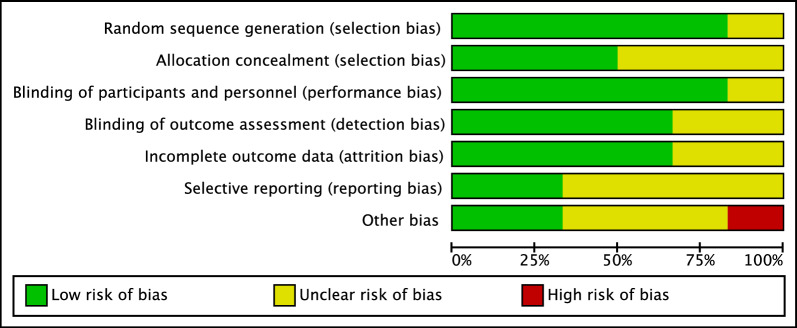
Cochrane risk of bias tool

The Cochrane risk of bias tool was performed to investigate the risk of bias of RCTs. Given the high quality of the included studies, the risk of selection bias was low. Most studies performed assessors blinding; therefore, the risk of detection bias was also low. The risk of attrition and reporting biases were moderate to low, as was the risk of other biases. Concluding, the risk of bias graph evidenced a good quality of the methodological assessment of RCTs (Fig. 2).

### Study characteristics and results of individual studies

Data from 31,705 patients were extracted. Of them, 62% (19,824) were women. The mean length of follow-up was 2.6 ± 0.8 years. The mean age of the patients was 68.7 ± 7.0 years, and the mean BMI was 27.5 ± 1.9 kg/m^2^. ANOVA found no statistically significant differences in age (*P* = 0.6), sex ratio (*P* = 0.4), and BMI (*P* = 0.8), attesting good baseline comparability of patient demographics. The generalities and demographics of the included studies are presented in Table 1.

**Table 1:** Generalities and patient baseline of the included studies (*FU* follow-up)

Author and year	Journal	Last FU (months)	Drug	Daily administration	Patients (*n*)	Mean age	Women (*%*)	Mean BMI (*kg/m*^*2*^)
Anderson et al. 2018 [16]	*N Engl J Med*	3	Aspirin	100	1707	63	51%	27
			Rivaroxaban	20	1717	63	50%	28
Eriksson et al. 2001 [37]	*Orthopedics*	2	Fondaparinux	2.5	849	65	70%	27
			Enoxaparin	40	862	65	67%	27
Eriksson et al. 2004 [38]	*J Thumb Haemost*	3	Ximelagatran	2	1377	67	65%	27
			Enoxaparin	40	1378	67	64%	27
Eriksson et al. 2006 [39]	*J Thumb Haemost*	2	Rivaroxaban	2.5	135	64	64%	28
			Rivaroxaban	5	139	65	54%	39
			Rivaroxaban	10	138	65	62%	28
			Rivaroxaban	20	137	66	57%	28
			Rivaroxaban	30	37	64	59%	29
			Enoxaparin	40	132	80	59%	28
Eriksson et al. 2006 [40]	*Circulation*	2	Rivaroxaban	5	128	84	56%	27
			Rivaroxaban	10	142	79	63%	27
			Rivaroxaban	20	139	69	59%	27
			Rivaroxaban	30	142	77	51%	27
			Rivaroxaban	40	137	80	59%	27
			Enoxaparin	40	157	80	64%	27
Eriksson et al. 2007 [41]	*Thromb Res*	2	Rivaroxaban	20	68	67	65%	27
			Enoxaparin	40	162	66	54%	28
			Rivaroxaban	5	76	64	62%	28
			Rivaroxaban	10	80	67	64%	28
			Rivaroxaban	30	88	66	58%	28
			Rivaroxaban	60	74	64	54%	28
			Rivaroxaban	40	77	66	58%	28
Eriksson et al. 2008 [42]	*N Engl J Med*	3	Rivaroxaban	10	2209	70	55%	28
			Enoxaparin	40	2224	69	54%	28
Eriksson et al. 2011 [43]	*BMJ*	3	Dabigatran	220	1010	72	53%	27
			Enoxaparin	40	1003	72	50%	27
Fuji et al. 2014 [44]	*J Arthoplasty*	3	Edoxaban	15	78	61	80%	26
			Edoxaban	30	72	60	95%	26
			Enoxaparin	40	74	58	79%	26
Fuji et al. 2015 [45]	*Thromb J*	3	Edoxaban	30	220	62	86%	25
			Enoxaparin	20	212	62	85%	25
Kakkar et al. 2008 [46]	*Lancet*	1	Rivaroxaban	10	1228	70	54%	26
			Enoxaparin	40	1229	71	53%	27
Lassen et al. 2010 [47]	*N Engl J Med*	4	Apixaban	5	2708	60	52%	28
			Enoxaparin	40	2699	60	53%	28
Lassen et al. 2002 [56]	*Lancet*	2	Fondaparinux	2.5	1140	66	57%	26
			Enoxaparin	40	1130	67	58%	27
Rascob et al. 2012 [49]	*J Bone Joint Surg Br*	3	Apixaban	5	2708	61	53%	28
			Enoxaparin	40	2699	61	54%	28

### Synthesis of results

**Fig. 3:**
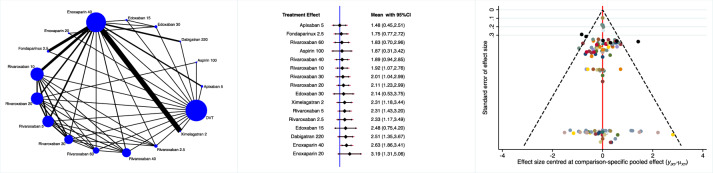
Edge, interval and funnel plots of the comparison: rate of DVT

Apixaban 5 mg, fondaparinux and rivaroxaban 60 mg were the most effective drugs in reducing the rate of DVT (Fig. 3).

**Fig. 4:**
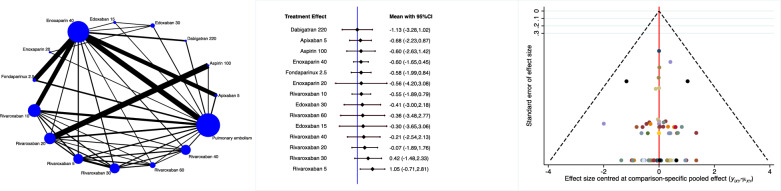
Edge, interval and funnel plots of the comparison: rate of PE

Dabigatran 220 mg, apixaban 5 mg and aspirin 100 mg were more effective in reducing the rate of PE (Fig. 4).

**Fig. 5:**
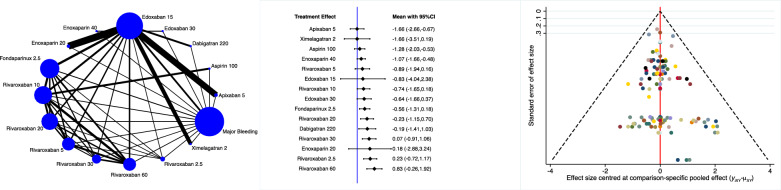
Edge, interval and funnel plots of the comparison: rate of major haemorrhages

Apixaban 5 mg, ximelagatran 2 mg and aspirin 100 mg were associated with the lowest rate of major haemorrhages (Fig. 5).

**Fig. 6:**
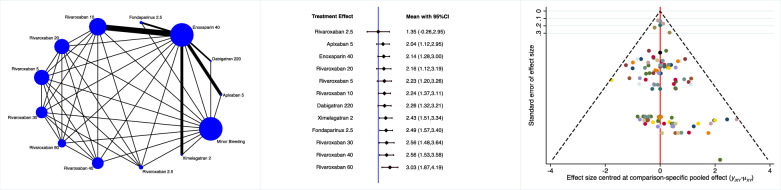
Edge, interval and funnel plots of the comparison: rate of minor haemorrhages

Rivaroxaban 2.5 mg, apixaban 5 mg and enoxaparin 40 mg were associated with the lowest rate of minor haemorrhages (Fig. 6).

## Discussion

According to the main findings of the present Bayesian network meta-analysis, the administration of apixaban 5 mg following THA demonstrated the best balance between VTE prevention and haemorrhage control.

Low-molecular-weight heparins (LMWHs) have been considered the standard of care for VTE prophylaxis after total hip arthroplasty given their low rate of heparin-induced thrombocytopaenia when compared with unfractionated heparin [57]. However, a relevant number of VTEs was evidenced after arthroplasty despite the use of LMWHs such as enoxaparin [58, 59]. NOACs emerged on the market approximately two decades ago, and do not necessitate routine coagulation monitoring. Promising results from the original studies [60, 61] regarding the benefit/risk ratio of NOACs prompted a series of high-quality RCTs regarding different types of prophylaxis and protocols for the prevention of VTE after THA [11, 16, 17, 19, 34, 37–41, 43–47, 49, 56]. Additionally, the Swedish Arthroplasty Registry reported that patients undergoing unilateral THA who received NOACs demonstrated a statistically significant lower rate of VTE compared with those who received low-molecular-weight heparin (LMWH) [62]. However, to date no consensus on the optimal prophylaxis for VTE following THA has been reached. Enoxaparin binds to and enhances the activity of antithrombin III (AT-III), and was the most commonly used prophylaxis before the introduction of the NOACs. AT-III is a physiological inhibitor of the coagulation process which inhibits the coagulation factors Xa and IIa (thrombin), but also factors XIa and IXa. Enoxaparin has also been reported to lead to an AT-III-dependent inhibition of factor VIIa, the induction of endogenous tissue factor pathway inhibitor (TFPI) and the reduction of von Willebrand factor (vWF) by the vascular endothelium [63, 64]. The pentasaccharide fondaparinux is a heparinoid which also indirectly inhibits factor Xa through the activation of AT-III. Fondaparinux can thus rapidly and selectively inhibit coagulation and has a relatively long half-life which allows the drug to obtain an antithrombotic effect for 24 h [32, 65]. Several NOACs are used as prophylaxis: apixaban (half-time around 12 h), edoxaban (half-time 10-14 h), and rivaroxaban (half-time 9 h). These three substances are reversible and highly selective for the inhibition of the factor Xa. Factor Xa is involved in the formation of factor IIa (thrombin) which catalyses fibrinogen into fibrin, inhibiting clotting [66]. Among these substances, rivaroxaban was the first orally administered direct inhibitor of factor Xa. In contrast to other indirect factor Xa inhibitors, such as fondaparinux or heparin, direct factor Xa inhibitors act on both free and clot-bound factor Xa and prothrombinase activity, prolonging clotting times [67–69]. Factor Xa inhibitors have no direct effect on platelet aggregation, but they only indirectly inhibit thrombin-induced platelet aggregation [66, 70]. Dabigatran is a NOACs which inhibits factor IIa (thrombin) [71]. Dabigatran reversibly binds the active site of thrombin, preventing thrombin-mediated activation of clotting factors. Dabigatran also inhibits tissue factor-induced platelet aggregation in plasma, demonstrating a greater inhibitory effect than the factor Xa inhibitors rivaroxaban and apixaban [69, 71]. Ximelagatran also binds factor IIa, and is activated into melagatran in the liver. Melagatran directly inhibits the serine protease alpha-thrombin, thus preventing thrombus formation. However, given their liver toxicity, ximelagatran and melagatran were withdrawn from the market in 2006.

All previously discussed substances affect plasmatic coagulation. Aspirin, in contrast, has an inhibitory effect on platelet aggregation through irreversibly blocking prostaglandin-endoperoxide synthase (also known as platelet cyclooxygenase, COX) at the functionally amino acid serine 530, which in turn irreversibly blocks thromboxane A_2_ formation in platelets [72].

In the present Bayesian network meta-analysis, the administration of apixaban 5 mg demonstrated the best balance between VTE prevention and haemorrhage control following THA. Lassen et al. [47] compared more than 5400 patients using apixaban 2.5 mg twice daily (*N* = 2708) and enoxaparin 40 mg daily (*N* = 2699) as prophylaxis after THA. Apixaban showed an absolute risk reduction of 2.5% in the composite outcome of either asymptomatic or symptomatic DVT, nonfatal PE, or death from any cause during the treatment period when compared with enoxaparin [47]. At the same time, no increased risk of major bleeding was observed [47]. Similarly, edoxaban was superior to enoxaparin in preventing VTE after THA without increasing the risk of major bleeding. A dose-dependent effect was thereby reported on the rate of major postoperative bleeding [73, 74]. In a phase II study, Fuji et al. [44] compared 264 patients receiving either edoxaban 15 mg or 30 mg daily or enoxaparin 20 mg twice daily after THA, observing comparable outcomes [44]. Edoxaban-induced prolongation of prothrombin time, international normalised ratio (INR) and activated partial thromboplastin time were proportional to plasma edoxaban concentration [44]. In the following phase III study including more than 600 patients, daily administration of endoxaban 30 mg resulted more effective than enoxaparin 2000 IU subcutaneously twice daily, without an increased rate of haemorrhagic events [45]. Two RCTs compared 2.5 mg of daily fondaparinux with 40 mg of daily enoxaparin for the prevention of VTE after THA [37, 56]. In one study, 1711 patients were evaluated after THA following a fracture of the hip; another study included 2270 patients after elective THA. Both RCTs demonstrated a relative reduction risk for VTE of more than 50% for fondaparinux, without an increased risk of clinically relevant postoperative bleeding [37, 56]. Rivaroxaban was evaluated in a dose-dependent fashion (2.5 mg, 5 mg, 10 mg, 20 mg, or 30 mg daily) in an RCT of 720 patients versus enoxaparin 40 mg daily [39]. The incidence of VTEs following rivaroxaban was comparable to that of enoxaparin [39]. A slight increase in postoperative bleeding major was observed with increasing doses of rivaroxaban [39]. In another phase IIa study, the administration of 2.5–30 mg twice daily or 30 mg once daily of rivaroxaban and enoxaparin 40 mg twice daily were compared [41]. The incidence of DVTs decreased in a dose-dependent manner with rivaroxaban down to the dosage of 20 mg, whereafter the rate increased again [41]. VTEs were lower in the 10 mg rivaroxaban group than with enoxaparin [41]. Major bleeding also increased in a dose-dependent manner, with rivaroxaban up to 10% [41]. In a large phase III RCT of 4433 patients, rivaroxaban 10 mg was compared with enoxaparin 40 mg daily [34]. The absolute risk reduction for major VTE was 1.7% and 2.6% for the composite outcome of DVT, nonfatal PE or death from any cause within 36 days after surgery in the patients allocated to receive rivaroxaban. Haemorrhages, although three times more frequent in the rivaroxaban group, were not statistically significantly different between the two groups [34]. Similar findings were observed by Kakkar et al. [75]. All three studies analysed direct factor Xa inhibitors, and demonstrated a relevant risk reduction for VTEs when the proper dosage was applied with no clinically relevant increase in haemorrhages [75]. A daily administration of dabigatran 220 mg was investigated in 2055 patients and compared with daily enoxaparin 40 mg. Dabigatran was superior in VTE but non-inferior to parenteral enoxaparin for the prevention of VTE and all-cause mortality after total hip arthroplasty [43]. No significant difference was observed in haemorrhages and other adverse events [43].

One study compared 5 days of prophylaxis using 81 mg daily of aspirin versus 10 mg daily of rivaroxaban in 1804 patients. Thereafter, patients were randomly assigned to continue rivaroxaban or to switch to aspirin for an additional 30 days after THA. After 90 days, no between groups difference was observed in VTE, PE or haemorrhagic events [16]. Usually, aspirin is considered to be suitable for the secondary prevention of arterial vascular events. Its role in the primary or secondary prophylaxis of VTE is controversial, especially in Continental and Southern Europe. In the present Bayesian meta-analysis, no difference between aspirin and other established drugs such as rivaroxaban was found. These findings are also consistent with findings from studies analysing the effect of aspirin after TKA [76–78].

Among the total extracted patients, rivaroxaban, enoxaparin and apixaban are much more represented compared with patients undergoing prophylaxis with alternative therapies. Indeed, 42% (12,791 of 30,371) of patients received enoxaparin, 23% (6891 of 30,371) rivaroxaban, 14% (4236 of 30,371) apixaban, 7% (1989 of 30,371) fondaparinux, 6% (1707 of 30,371) aspirin, 5% (1377 of 30,371) ximelagatran, 3% (1010 of 30,371) dabigatran and 1% (370 of 30,371) edoxaban. These differences in sample size can impact the reliability of the results of the present study. Moreover, there was high heterogeneity in the administration protocols, especially in the duration of the prophylaxis and relevant dosages. Given these heterogeneities, results should be considered with caution. Almost all studies investigated VTE prevention and haemorrhage control following primary THA. One study included patients who underwent THA in both primary and revision setting [16]. However, more than 90% of these patients underwent primary THA. Whether primary or revision setting influence VTE and haemorrhage in THA is unclear.

Further studies are warranted to validate the results of the present study in a clinical setting. The mean length of the follow-up was 2–3 months in most studies. By that time, patients should have reached full weight bearing and almost regular activity levels. The dosage of the prophylaxis may be influenced by therapeutic intent, body weight and individual pharmacological clearance (e.g. estimated glomerular filtration rate). Future studies should investigate combinations of anticoagulants with an individual risk-stratified dosage. Of note, the results from RCTs usually apply a strict protocol; however, factors such as patient compliance might thus not be reflected in the results. The individual risk profile of simple patients was also not taken into account. Moreover, when deciding the anticoagulant prophylaxis to be implemented, other factors might still be considered; for example, the possibility to antagonise the drug in those rare cases of excessive bleeding. While such antagonisation is possible to at least 50% with protamine sulphate for enoxaparin [79], for NOACs it is hardly available and/or very expensive.

## Conclusion

Daily administration of apixaban 5 mg resulted in the best prevention of thromboembolic events and appropriate control of the risk of haemorrhages following THA.

## Data Availability

The datasets generated during and/or analysed during the current study are available throughout the manuscript.
